# Type of preoperative therapy and stage-specific survival after surgery for rectal cancer: a nationwide population-based cohort study

**DOI:** 10.1007/s00428-019-02638-1

**Published:** 2019-08-28

**Authors:** Steven L. Bosch, Rob H. A. Verhoeven, Valery E. P. P. Lemmens, Femke Simmer, Philip Poortmans, Johannes H. W. de Wilt, Iris D. Nagtegaal

**Affiliations:** 1 Department of Pathology, Radboud University Medical Center, P.O. Box 9101, 6500 HB Nijmegen, The Netherlands; 2 Netherlands Comprehensive Cancer Organization/Netherlands Cancer Registry, P.O. Box 19079, 3501 DB Utrecht, The Netherlands; 3 Department of Public Health, Erasmus MC University Medical Centre, P.O. Box 2040, 3000 CA Rotterdam, The Netherlands; 4 Department of Radiation Oncology, Institut Curie, 26 Rue d’Ulm, 75248 Paris Cedex 05, France; 5 Department of Surgery, Radboud University Medical Center, Nijmegen, The Netherlands

**Keywords:** Rectal cancer, pTNM, ypTNM, Downstaging, Preoperative therapy, TNM stage, Survival

## Abstract

Preoperative chemoradiation therapy (CRT) may induce downstaging in rectal cancer (RC). Short-course radiation therapy (SC-RT) with immediate surgery does not cause substantial downstaging. However, the TNM classification adds the “y” prefix in both groups to indicate possible treatment effects. We aim to compare stage-specific survival in these patients. RC patients treated with surgery only, preoperative SC-RT followed by surgery within 10 days, or preoperative CRT, and diagnosed between 2008 and 2014 were included in this population-based study. Clinicopathological and outcome characteristics were analyzed. The study included 11,925 patients. Large discrepancies existed between clinical and pathological stages after surgery only. Surgery-only patients were older with more comorbidities compared with SC-RT and CRT and had worse 5-year survival (64%, 76%, and 74%, respectively; *p* < 0.001). Five-year survival for stage I was similar after CRT and SC-RT (85% vs. 85%; *p* = 0.167) and comparable between CRT-treated patients with stage I and those reaching a pathological complete response (pCR; 85% vs. 89%; *p* = 0.113). CRT was independently associated with worse overall survival compared with SC-RT for stage II (HR 1.57 [95%CI 1.27–1.95]; *p* < 0.001) and stage III (HR 1.43 [95%CI 1.23–1.70]; *p* < 0.001). Stage I disease after CRT has an excellent prognosis, comparable with pCR and with same-stage SC-RT-treated patients without regression. Stage II or III after CRT has worse prognosis than after SC-RT with immediate surgery. TNM should take the impact of preoperative therapy type on stage-specific survival into account. In addition, clinical stage was a poor predictor of pathological stage.

## Introduction

The standard of care for rectal cancer (RC) patients is total mesorectal excision (TME) with or without preoperative therapy depending on clinical stage. For patients with locally advanced RC, preoperative treatment consists of chemoradiation therapy (CRT) intended to reduce local recurrence rates and to facilitate radical surgery by inducing tumor regression and possible downstaging [1, 2]. Significant tumor and nodal downstaging is reported in patients treated with CRT and depends on factors such as tumor type, clinical stage, and interval between radiation therapy and surgery [3–7]. Downstaging is associated with an improved prognosis, especially in 8–24% of patients with a pathological complete response (pCR) [1, 6, 8, 9]. The TNM staging system [10, 11] recommends adding the prefix “y” to the TNM stage after preoperative therapy to indicate that a tumor may have undergone treatment-induced response or regression.

Especially in Western European countries, patients may also undergo preoperative short-course radiation therapy (SC-RT) followed by immediate surgery [12–15]. Randomized trials show a small downstaging effect in these patients [13, 14], and according to the definitions of the TNM classification, the “y” prefix should be added. However, downstaging does not occur after SC-RT if the overall treatment time (i.e., interval between start of radiation therapy and rectal resection) does not exceed 10 days [16]. The prognostic significance of ypTNM stage for patients in these groups (with vs. without possible downstaging) is still unclear. Due to the differences in levels of downstaging between groups of patients treated either with preoperative SC-RT followed by immediate surgery or with CRT [17], it may be hypothesized that the prognostic implications of the “y” prefix depend on the type of preoperative therapy received, which limits the prognostic value of staging.

The purpose of this study is therefore to investigate on a population level whether stage-specific overall survival is different between patients treated with either SC-RT followed by surgery within 10 days after start of treatment (no tumor regression expected; ypTNM by definition, but may reflect pTNM) or preoperative long-course CRT (intended to induce tumor regression; ypTNM) and to compare results with patients who underwent surgery only (pTNM).

## Patients and methods

### Study design and patient selection

A population-based approach was employed using data from the nationwide Netherlands Cancer Registry (NCR). This institute collects data on all newly diagnosed cancer patients in the Netherlands since 1989. The registration is primarily based on notification by the Dutch national digital pathology registry (PALGA). Patient and clinicopathological data are routinely collected from medical records by specially trained data managers. Tumor location and histology is registered according to the ICD-O3 classification. Follow-up data and vital status are retrieved by linkage to the nationwide population registries network.

Patients with RC diagnosed between January 2008 and December 2014 who underwent a surgical resection were selected from the NCR. Clinicopathological characteristics and overall survival (including TNM stage-specific survival) were compared between patients treated with surgery only, preoperative SC-RT with an overall treatment time that did not exceed 10 days, and preoperative long-course CRT. The maximum interval of 10 days between start of SC-RT and surgery was chosen, since tumor regression is not likely to occur within this timeframe [16]. For patients in the CRT group, an interval of at least 63 days (duration of CRT + 4 weeks to provide the opportunity for tumor regression) and no longer than 182 days (6 months; arbitrary) was required.

Cases were excluded if the date of surgery was not available or if there was presence of distant metastases at time of surgery or missing data regarding distant metastases. The same was true for cases with histopathological tumor type other than adenocarcinoma, mucinous carcinoma, or signet ring cell carcinoma. Other exclusion criteria were missing values for pathological T or N categories and surgical procedures other than a low anterior resection (LAR), Hartmann’s procedure, abdominoperineal excision (APE), or intersphincteric resection.

Comorbidity was only registered in the NCR for one specific region in the Netherlands, covering 12% of the population. A subgroup analysis of this data was performed.

### Preoperative therapy

The prevailing RC clinical guideline [18] during the inclusion period recommended preoperative SC-RT for primarily resectable RC (with the exception of cT1N0 tumors) consisting of 5 × 5 Gy followed by surgery within 1 week. Long-course CRT consisting of 45–50 Gy given in 25 fractions of 1.8–2.0 Gy per day with concurrent oral chemotherapy (capecitabine 825–1000 mg/m^2^ twice daily) and followed by surgery within 4–6 weeks was indicated for locally advanced RC (i.e., patients with clinical N2 disease, cT4 tumors, or tumors with suspected involvement of the mesorectal fascia on imaging). Patients treated with surgery only either had cT1N0 disease or were unfit or did not give consent to undergo preoperative treatment.

### Statistical analysis

All data was entered in a database and analyzed using IBM SPSS Statistics for Windows, version 22.0, Armonk, NY: IBM Corp. Categorical variables were analyzed using the *χ*^2^ test. Cumulative overall survival was analyzed using the Kaplan-Meier method with log rank test. Univariable and multivariable analyses were performed by entering all applicable clinical and pathological factors in a Cox regression model. A *p* value of < 0.05 was considered statistically significant whereas a *p* value of < 0.1 was taken to reflect a trend towards significance.

## Results

### Patient selection

**Fig. 1:**
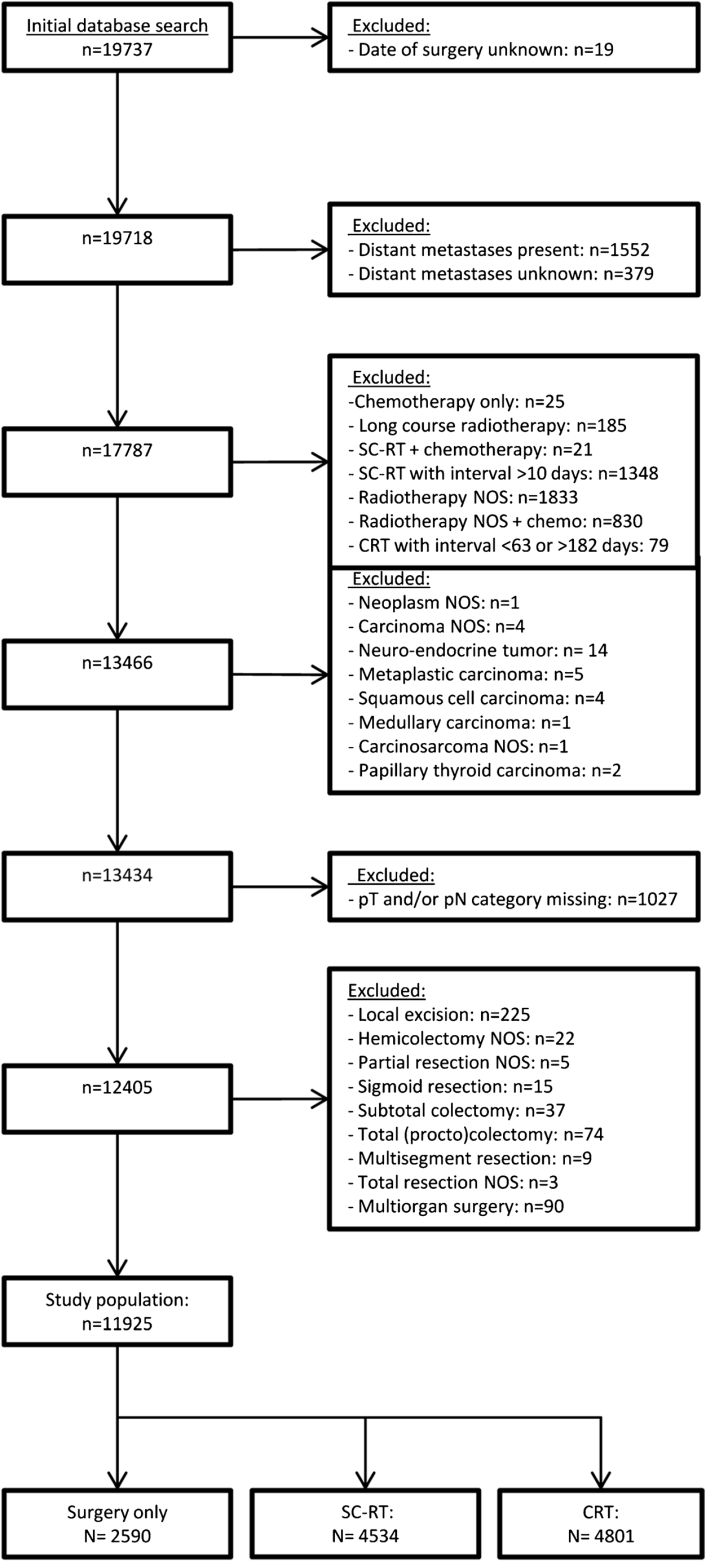
Flow diagram showing the selection process. SC-RT, short-course radiation therapy; CRT, chemoradiation therapy; NOS, not otherwise specified; pT, pathological tumor category; pN, pathological nodal category

The initial search of the NCR database identified 19,737 patients. Figure 1 depicts the selection process, resulting in the inclusion of a total of 11,925 patients (2590 with surgery only, 4534 with SC-RT, and 4801 with CRT).

### Clinicopathological factors

Table 1 provides the clinical and pathological characteristics. Median interval between start of SC-RT and surgery was 8 days (range 0–10), and in the CRT group, the median interval was 100 days (range 63–182). Patients in the surgery-only group were older than those in the SC-RT group, who in turn were older than the CRT-treated patients (age > 75 years; 40%, 28%, and 13% in the surgery-only, SC-RT, and CRT groups, respectively; *p* < 0.001). Other factors that were significantly associated with the type of preoperative treatment were cT category, cN category, cTNM stage, type of resection, pT category, pN category, pTNM stage, CRM involvement, histological type, and presence of postoperative chemotherapy. Patients in the CRT group showed ypT0 in 20% of cases and had a reduced rate of (y)pT2 and (y)pT3 tumors compared with those in the surgery-only and SC-RT groups (*p* < 0.001). A pCR (ypT0N0) occurred in 18% of cases after CRT.

**Table 1:** Clinicopathological characteristics of patients treated with surgery only, SC-RT, and CRT

	Total		Surgery only		SC-RT		CRT		*p* value
	*n*	%	*n*	%	*n*	%	*n*	%	
Total	11,925		2590		4534		4801		
Gender									0.092
Male	7610	63.8	1608	62.1	2899	63.9	3103	64.6	0.485^a^
Female	4315	36.2	982	37.9	1635	36.1	1698	35.4	
Age at diagnosis									< 0.001
0–44	342	2.9	48	1.9	89	2.0	205	4.3	
45–59	2583	21.7	334	12.9	886	19.5	1363	28.4	
60–74	6078	51.0	1171	45.2	2289	50.5	2618	54.5	
75+	2922	24.5	1037	40.0	1270	28.0	615	12.8	
Clinical T category									< 0.001
cT1	302	3.1	147	8.0	133	3.8	22	0.5	
cT2	2656	26.9	800	43.7	1415	40.1	441	9.8	
cT3	6070	61.4	783	42.8	1933	54.7	3354	74.2	
cT4	852	8.6	99	5.4	52	1.5	701	15.5	
Missing	2045		761		1001		283		
Clinical N category									< 0.001
cN0	5272	49.7	1824	80.7	2594	65.9	854	19.4	
cN1	3284	31.0	348	15.4	1193	30.3	1743	39.6	
cN2	2043	19.3	88	3.9	148	3.8	1807	41.0	
Missing	1326		330		599		397		
Clinical TNM stage									< 0.001
Stage I	1879	20.2	789	45.2	989	30.3	109	2.3	
Stage II	2117	22.7	519	29.8	930	28.5	686	15.5	
Stage III	5327	57.1	436	25.0	1341	41.1	3602	82.2	
Missing	2602		846		1274		482		
Type of resection									< 0.001
Sphincter saving^b^	8161	68.4	2152	83.1	3361	74.1	2648	55.2	
Non-sphincter saving^c^	3764	31.6	438	16.9	1173	25.9	2153	44.8	
Pathological T category									< 0.001
(y)pT0	979	8.2	5	0.2	34	0.7	940	19.6	
(y)pT1	1050	8.8	380	14.7	336	7.4	334	7.0	
(y)pT2	4073	34.2	893	34.5	1852	40.8	1328	27.7	
(y)pT3	5450	45.7	1206	46.6	2237	49.3	2007	41.8	
(y)pT4	373	3.1	106	4.1	75	1.7	192	4.0	
Pathological N category									< 0.001
(y)pN0	8098	67.9	1775	68.5	2958	65.2	3365	70.1	
(y)pN1	2632	22.1	568	21.9	1081	23.8	983	20.5	
(y)pN2	1195	10.0	247	9.5	495	10.9	453	9.4	
Pathological TNM stage									< 0.001
Stage 0	895	7.5	4	0.2	25	0.6	866	18.0	
Stage I	4070	34.1	1058	40.8	1705	37.6	1307	27.2	
Stage II	3133	26.3	713	27.5	1228	27.1	1192	24.8	
Stage III	3827	32.1	815	31.5	1576	34.8	1436	29.9	
CRM involvement^d^									< 0.020
Absent	9605	92.4	1981	91.6	3695	93.3	3929	92.0	0.018*
Present	788	7.6	181	8.4	264	6.7	343	8.0	
Missing	1532		428		575		529		
Histological type									0.009
Adenocarcinoma	11,049	92.7	2436	94.1	4208	92.8	4405	91.8	0.158*
Mucinous carcinoma	825	6.9	144	5.6	308	6.8	373	7.8	
Signet ring cell carcinoma	51	0.4	10	0.4	18	0.4	23	0.5	
Postoperative CTx									0.013
No	11,175	93.7	2395	92.5	4263	94.0	4517	94.1	
Yes	750	6.3	195	7.5	271	6.0	284	5.9	

*SC-RT* short-course radiation therapy, *CRT* chemoradiation therapy, *CRM* circumferential resection margin, *CTx* chemotherapy

^a^SC-RT vs. CRT

^b^Includes 6887 patients with low anterior resection, 1186 patients with Hartmann’s procedure, and 88 patients with intersphincteric resection

^c^Patients with abdominoperineal excision

^d^Circumferential resection margin (CRM) involvement was defined as tumor distance to the CRM ≤ 1 mm

A subgroup analysis of cases with available comorbidity data (*n* = 1270) showed a higher rate of comorbidities in the surgery-only group compared with both the SC-RT and CRT groups with ≥ 2 comorbidities in 45%, 35%, and 25% of cases for surgery only, SC-RT, and CRT, respectively (*p* < 0.001 for surgery only vs. CRT; *p* = 0.006 for surgery only vs. SC-RT; *p* = 0.003 for SC-RT vs. CRT).

### Correlation between cTNM and (y)pTNM

The data showed substantial discrepancies between clinical and pathological TNM stages (Table 2). Patients with suspected LN on clinical imaging had histopathology showing nodal disease in 57%, 49%, and 35% of cases that were treated with surgery only, SC-RT, and CRT, respectively (*p* < 0.001). Patients with clinical stage I disease had histopathological nodal involvement in 19%, 24%, and 13% after surgery only, SC-RT, and CRT, respectively (*p* = 0.004). For patients with clinical stage II, this was 26%, 30%, and 15%, respectively (*p* < 0.001). In the CRT group, there was complete tumor regression in 30%, 19%, and 17% of cases for patients with clinical stage I, II, and III disease, respectively (*p* < 0.001).

**Table 2:** Correlation between clinical and pathological stages

*N* = 9323^a^		Clinical stage					
		Stage I		Stage II		Stage III	
		*n*	%	*n*	%	*n*	%
Surgery only	Pathological stage						
	Stage 0	2	0.3	1	0.2	0	0.0
	Stage I	481	61.0	146	28.1	81	18.6
	Stage II	154	19.5	237	45.7	108	24.8
	Stage III	152	19.3	135	26.0	247	56.7
	Total	789	100.0	519	100.0	436	100.0
SC-RT	Pathological stage						
	Stage 0	15	1.5	1	0.1	4	0.3
	Stage I	535	54.1	286	30.8	359	26.8
	Stage II	199	20.1	368	39.6	320	23.9
	Stage III	240	24.3	275	29.6	658	49.1
	Total	989	100.0	930	100.0	1341	100.0
CRT	Pathological stage						
	Stage 0	30	29.7	129	19.3	601	16.9
	Stage I	40	39.6	205	30.7	898	25.3
	Stage II	18	17.8	235	35.2	821	23.1
	Stage III	13	12.9	99	14.8	1230	34.6
	Total	101	100.0	668	100.0	3550	100.0

*SC-RT* short-course radiation therapy, *CRT* chemoradiation therapy

^a^Cases with missing values for clinical TNM stage were excluded (*n* = 2602)

### Survival analysis

Median follow-up was 28 months (range 0–84 months) and 1949 deaths were recorded (16.3%). Cumulative 5-year overall survival was 73% (Table 3). The surgery-only group showed worse overall survival than the SC-RT and CRT groups (64%, 76%, and 74% for surgery only, SC-RT, and CRT, respectively; *p* < 0.001). However, there was no significant difference between patients treated with SC-RT vs. CRT (*p* = 0.147).

**Table 3:** Overall survival

	Cumulative 5-year overall survival	*p* value^a^	HR	95%CI
Overall	73%			
Preoperative therapy		< 0.001		
SC-RT	76.3	0.147^†^	1.00	–
CRT	73.5		1.07	0.96–1.19
None (surgery only)	64.1		1.73	1.55–1.94
Gender		< 0.001		
Male	71.3		1.00	–
Female	76.0		0.77	0.70–0.85
Age at diagnosis		< 0.001		
0–44	85.2		1.00	–
45–59	82.0		1.18	0.81–1.72
60–74	76.9		1.76	1.23–2.52
75+	56.1		4.10	2.87–5.87
Clinical T category		< 0.001		
cT1	75.0		1.00	–
cT2	78.7		0.99	0.70–1.39
cT3	73.1		1.36	0.98–1.89
cT4	61.0		2.15	1.51–3.05
Missing	71.2		1.52	1.09–2.12
Clinical N category		0.007		
cN0	74.2		1.00	–
cN1	71.9		1.10	0.99–1.23
cN2	73.6		0.98	0.84–1.13
Missing	70.6		1.22	1.08–1.38
Clinical TNM stage		< 0.001		
Stage I	78.9		1.00	–
Stage II	72.0		1.46	1.23–1.73
Stage III	72.4		1.38	1.18–1.61
Missing	71.5		1.51	1.29–1.77
Type of resection		0.004		
Sphincter saving	74.0		1.00	–
Non-sphincter saving	71.1		1.15	1.05–1.26
Pathological T category		< 0.001		
pT0	87.2		0.73	0.54–0.99
pT1	82.0		1.00	–
pT2	81.0		1.14	0.92–1.41
pT3	65.2		2.22	1.81–2.71
pT4	45.9		4.50	3.48–5.82
Pathological N category		< 0.001		
pN0	79.2		1.00	–
pN1	67.2		1.65	1.49–1.84
pN2	47.6		3.13	2.79–3.51
Pathological stage		< 0.001		
Stage 0	89.2		0.62	0.47–0.83
Stage I	83.3		1.00	–
Stage II	71.5		1.76	1.55–2.00
Stage III	60.9		2.61	2.32–2.93
Histological type		< 0.001		
Adenocarcinoma	73.9		1.00	–
Mucinous carcinoma	66.2		1.41	1.21–1.63
Signet ring cell carcinoma	21.9		4.17	2.74–6.35
CRM involvement		< 0.001		
Absent	75.0		1.00	–
Present	53.5		2.24	1.97–2.56
Missing	73.1		1.17	1.04–1.32
Postoperative CTx		0.007		
No	72.7		1.00	–
Yes	77.4		0.78	0.65–0.93

*SC-RT* short-course radiation therapy, *CRT* chemoradiation therapy, *CRM* circumferential resection margin, *CTx* chemotherapy

^a^Log-rank test

^†^SC-RT vs. CRT

Clinical stage before neoadjuvant therapy was significantly associated with overall survival, although the hazard ratios are lower than those for pathological stage, and in the study population, the hazard ratios do not consistently increase with clinical stage in contrast to pathological stage, suggesting that the prognostic value of clinical stage is limited (HR 1.00, HR 1.46, and HR 1.38 for clinical TNM stages I, II, and III, respectively; and HR 0.62, HR 1.00, HR 1.76, and HR 2.61 for pathological TNM stages 0, I, II, and III, respectively).

**Fig. 2:**
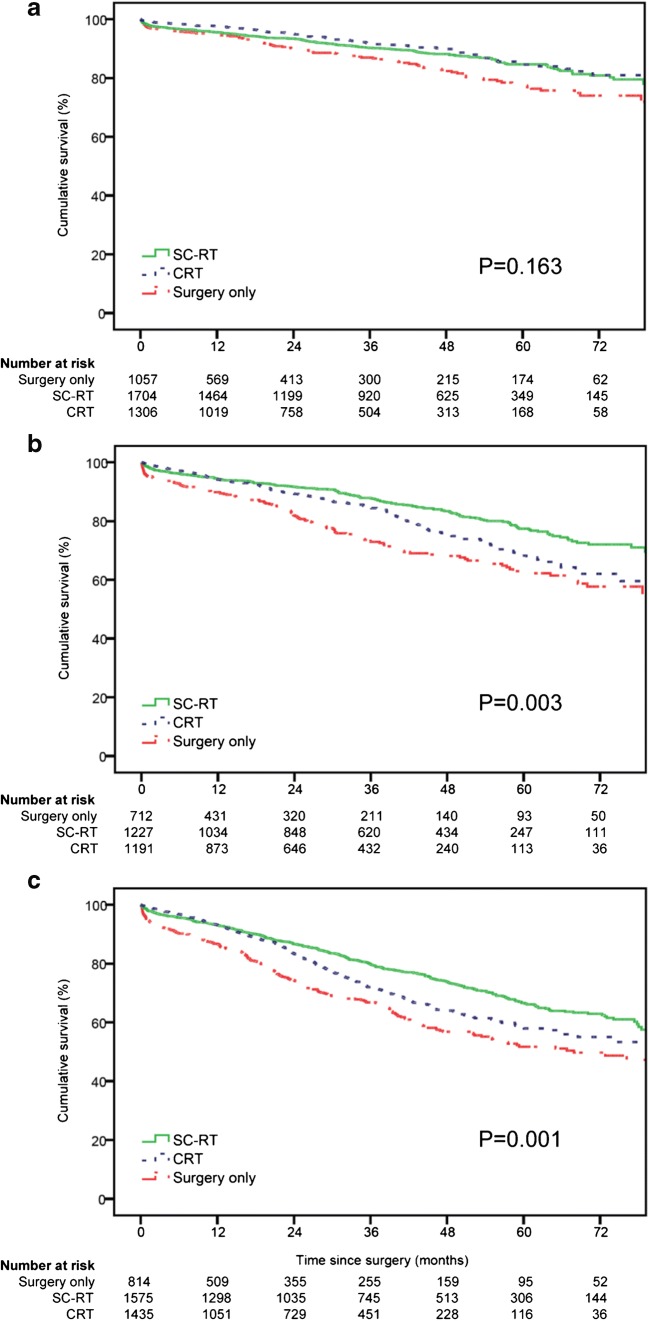
Overall survival for patients treated with surgery only, SC-RT, or CRT. **a** Pathological stage I. **b** Pathological stage II. **c** Pathological stage III. SC-RT, short-course radiation therapy; CRT, chemoradiation therapy

Figure 2a–c show stage-specific overall survival for patients with surgery only, SC-RT, and CRT. Survival was worst in the surgery-only group (cumulative 5-year survival 77%, 63%, and 52% for pathological stages I, II, and III, respectively; *p* ≤ 0.003 compared with SC-RT and CRT). SC-RT-treated patients with pathological stage I disease had similar overall survival as same-stage patients in the CRT group (cumulative 5-year survival 85% vs. 85%, respectively; *p* = 0.167). After CRT overall survival was comparable in patients with pathological stage I and those who reached a pCR (cumulative 5-year survival 85% vs. 89% respectively; *p* = 0.113). The SC-RT group showed better survival than the CRT group for patients with pathological stage II (cumulative 5-year survival 77% vs. 68% for SC-RT vs. CRT, respectively; *p* = 0.002) and stage III (cumulative 5-year survival 67% vs. 58% for SC-RT vs. CRT, respectively; *p* < 0.001).

The multivariable analysis (Table 4) showed that CRT was independently associated with a higher mortality compared with SC-RT in patients with pathological stage II (HR 1.57 [95%CI 1.27–1.95]; *p* < 0.001) and stage III (HR 1.43 [95%CI 1.23–1.70]; *p* < 0.001), but not in those with stage I (HR 0.99 [95%CI 0.77–1.27]; *p* = 0.146). The hazard ratio for patients with surgery only was also increased compared with SC-RT for patients with pathological stage II (HR 1.67 [95%CI 1.35–2.08]; *p* < 0.001) and stage III (HR 1.60 [95%CI 1.36–1.87]; *p* < 0.001), but not stage I (HR 1.25 [95%CI 0.99–1.59]; *p* = 0.137).

**Table 4:** Multivariable Cox regression analysis for overall survival of patients with pathological TNM stages I–III

	(y)pTNM stage I			(y)pTNM stage II			(y)pTNM stage III		
	HR	95%CI	*p* value	HR	95%CI	*p* value	HR	95%CI	*p* value
Preoperative therapy			0.146			< 0.001			< 0.001
SC-RT	1.00	–		1.00	–		1.00	–	
CRT	0.99	0.77–1.27		1.57	1.27–1.95		1.43	1.23–1.70	
None (surgery only)	1.25	0.99–1.59		1.67	1.35–2.08		1.60	1.36–1.87	
Gender			< 0.001			0.007			< 0.001
Male	1.00	–		1.00	–		1.00	–	
Female	0.61	0.49–0.76		0.78	0.65–0.94		0.77	0.67–0.89	
Age at diagnosis			< 0.001			< 0.001			< 0.001
0–44	0.81	0.24–2.71		0.80	0.37–1.74		0.97	0.61–1.53	
45–59	1.00	–		1.00	–		1.00	–	
60–74	2.99	1.95–4.59		1.23	0.94–1.61		1.35	1.12–1.63	
75+	7.88	5.14–12.10		2.89	2.20–3.79		2.81	2.29–3.45	
Type of resection			0.147			0.025			0.002
Sphincter saving	1.00	–		1.00	–		1.00	–	
Non-sphincter saving	1.17	0.95–1.45		1.24	1.03–1.49		1.25	1.09–1.44	
pT category			0.588			< 0.001			< 0.001
pT0	–	–		–	–		1.48	0.75–2.90	
pT1	1.00	–		–	–		1.00	–	
pT2	0.93	0.73–1.20		–	–		1.20	0.75–1.92	
pT3	–	–		1.00	–		1.91	1.22–3.00	
pT4	–	–		1.79	1.32–2.42		3.03	1.85–4.97	
pN category									< 0.001
pN1	–	–		–	–		1.00	–	
pN2	–	–		–	–		1.88	1.65–2.15	
Histological type			0.031			0.176			0.022
Adenocarcinoma	1.00	–		1.00	–		1.00	–	
Mucinous carcinoma	0.78	0.48–1.25		1.15	0.87–1.52		1.16	0.95–1.41	
Signet ring cell carcinoma	11.29	1.56–81.70		2.57	0.82–8.07		1.85	1.13–3.03	
CRM involvement			0.040			0.001			< 0.001
Absent	1.00	–		1.00	–		1.00	–	
Present	1.76	1.14–2.71		1.61	1.25–2.07		1.54	1.29–1.84	
Missing	1.03	0.80–1.33		1.23	0.98–1.54		1.15	0.96–1.38	
Postoperative CTx			0.474			0.008			< 0.001
No	1.00	–		1.00	–		1.00	–	
Yes	0.60	0.15–2.43		0.30	0.13–0.73		0.56	0.46–0.69	

*p* pathological, *SC-RT* short-course radiation therapy, *CRT* chemoradiation therapy, *AC* adenocarcinoma, *MC* mucinous carcinoma, *SRCC* signet ring cell carcinoma, *CRM* circumferential resection margin, *CTx* chemotherapy

## Discussion

In this population-based study, using data from the Netherlands Cancer Registry, long-term stage-specific survival data was analyzed from 11,925 RC patients who underwent TME surgery with or without preoperative treatment consisting of either SC-RT or CRT. Patients with downstaging to pathological stage I disease after CRT had an excellent 5-year overall survival which was similar as in SC-RT-treated patients without downstaging (85%). For patients with pathological stages II and III, survival was significantly worse after CRT (68% and 58%) compared with SC-RT (77% and 67%). In addition, clinical staging was a poor predictor of pathological stage based on the large discrepancies between clinical and pathological stages in the surgery-only group.

In the study period, the national clinical guideline [18] recommended preoperative SC-RT with 5 × 5 Gy and surgery within 1 week for primarily resectable RC (with the exception of cT1N0 tumors) and long-course CRT consisting of 45–50 Gy given in 25 fractions of 1.8–2.0 Gy per day with concurrent oral capecitabine for locally advanced RC (see “Patients and methods” section for details). Patients treated with surgery only in this period either had cT1N0 disease or were unfit or did not give consent to undergo the indicated preoperative therapy. Indeed, patients in the surgery-only group were found to be substantially older and had more comorbidity compared with both the SC-RT and CRT groups. These patients were therefore considered to be unsuitable as a control population in the current study.

On the other hand, the comparison of the SC-RT- and CRT-treated patients in this study yields some interesting results. Although some small downstaging effect on T-stage and nodal downstaging have been reported in randomized trials after SC-RT with immediate surgery [13, 14], the SC-RT-treated patients in the current study all had an overall treatment time not exceeding 10 days, and evidence from a large randomized controlled trial showed that tumor and nodal downstaging do not occur in this short timeframe [16]. Furthermore, stage-specific 10-year overall survival was shown to be similar in randomized patients with preoperative SC-RT and those with surgery only or surgery with selective postoperative CRT [12–14]. This lack of downstaging and absence of a survival difference suggest that the SC-RT-treated patients in the current study may be regarded best as pTNM rather than ypTNM [19].

The observed difference in stage-specific survival between patients treated with CRT (substantial downstaging) and SC-RT (no downstaging) was substantial and highly significant, and the effect was independent of several known possible confounders. However, selection bias may be a concern when interpreting these results. The inherently higher levels of treatment-induced toxicity caused by CRT may motivate clinicians to withhold this treatment in elderly patients with comorbidity, whereas the same comorbidity level would not preclude a treatment with much less toxic SC-RT. Unfortunately, comorbidity data was not available in the majority of patients and the results of the multivariable analysis could therefore not be corrected for this confounder. However, the subset analysis of patients with available comorbidity data showed that comorbidity levels were higher in the SC-RT than in the CRT group. As a consequence, the SC-RT group as a whole may be expected to show a bias towards a worse prognosis compared with the CRT group. However, the multivariable analysis showed the direct opposite with a better prognosis in the SC-RT group for patients with stage II and III disease. The observed survival differences may therefore be expected to be even larger if the results could be adjusted for comorbidity.

The introduction of preoperative CRT for RC which has resulted in tumor downstaging in a substantial proportion of patients has not resulted in improved survival compared with selective postoperative CRT [20]. The stage-specific outcome differences between the groups in this study are therefore not based on a therapeutic effect of the preoperative treatment, but are probably best explained by pathological stage migration. Patients with nodal disease may undergo sterilization of involved LN after CRT resulting in classification as pathological stage I or II disease instead of stage III.

Patients with downstaging to ypTNM stage I reflect a selection of treatment-sensitive tumors. The excellent survival observed in these patients, which is similar to SC-RT-treated pTNM stage I patients, suggests that potential occult disease or metastases also undergo substantial regression in patients with treatment-sensitive tumors. As a consequence, the observed mortality will increase in the ypTNM stage III patients with residual nodal metastases, since this group constitutes a selection of patients with treatment-resistant tumors. Furthermore, mortality can also be expected to increase in the ypTNM stage II group after CRT. This group is enriched with cases that had nodal disease at baseline, with higher risk of occult residual disease but with only intermediate regression to therapy, and this may correspond to intermediate regression of potential occult residual disease as well. This effect, called “the reverse of the Will Rogers phenomenon,” has been described before with data from the German CAO/ARO/AIO-94 trial, showing that patients with pathological stage II disease after preoperative CRT had worse overall survival than same-stage patients from the control arm treated with selective postoperative CRT [21].

A limitation to the current study is that it is not possible to determine the exact rate of stage migration in CRT-treated patients, since clinical staging (especially cN category) is notoriously unreliable [22–25]. Indeed, data from this study showed large discrepancies between clinical and pathological stages in the surgery-only group (no downstaging by definition) with pathologically confirmed LN metastases in only 57% of patients with clinical stage III disease. The differences between clinical and pathological stages in the surgery-only and SC-RT groups and at least a part of the variation observed in the CRT group are therefore probably related to the imprecision of clinical staging and not to actual stage migration. This imprecision is reflected in the relatively limited prognostic value of clinical stage (cTNM) compared with definitive pathological stage ((y)pTNM) observed in this study. Another important restriction is the lack of an adequate pTNM control group, due to the high level of selection bias in patients treated with surgery only.

Furthermore, precise details regarding chemotherapy types and doses received were not available for individual patients, since the national cancer registry does not document this information. It is likely that a part of individual patients in the long-course CRT group received doses that are not equal to the recommended dose due to toxicity issues. In addition, it is possible that some patients in this group received additional chemotherapeutic agents besides capecitabine as part of a clinical trial. Unfortunately, the impact of these minor treatment variations on the results of this study cannot be ascertained.

In conclusion, this population-based study provides evidence that pathological stage I after preoperative CRT for RC is associated with an excellent prognosis, which is comparable with reaching a pCR and similar to same-stage SC-RT-treated patients without tumor regression. In patients with pathological stage II and III disease after CRT, the prognosis is worse than after SC-RT with immediate surgery. These results contain important prognostic information for individual patients and physicians and may have consequences for predictive models. Staging systems, such as TNM, should therefore take stage-specific survival differences between patients treated with different preoperative therapy regimens into account.
